# Supplementary material for: Sea level rise from climate change is expected to increase the release of arsenic into Bangladesh’s drinking well water by reduction and by the salt effect
Source: PLoS One. 2024 Jan 17;19(1):e0295172. doi: 10.1371/journal.pone.0295172 (PMC10793926; doi:10.1371/journal.pone.0295172)
Supplement: S3 File — (DOCX) [file pone.0295172.s004.docx]

**The derivation of a pH-dependent Nernst equation. This derivation was confirmed by Marwan Said Abualrub, Ph.D., and Amrutaa Vibho.**

$$aA+mH^{+}\rightleftarrows bB \left( 4 \right)$$

$$E_{\mathrm{cell}}=E_{\mathrm{cell}}^{^{\circ}}-\frac{2.303RT}{\mathrm{nF}}\log_{10} \left( \frac{\left[ B \right]^{b}}{\left[ A \right]^{a}\left[ H^{+} \right]^{m}} \right)$$

$$\mathrm{Using}\log_{10} xy=\log_{10} x+\log_{10} y$$

$$E_{\mathrm{cell}}=E_{\mathrm{cell}}^{^{\circ}}-\left( \frac{2.303RT}{\mathrm{nF}}\log_{10} \left( \frac{\left[ B \right]^{b}}{\left[ A \right]^{a}} \right)+\frac{2.303RT}{\mathrm{nF}}\log_{10} \left( \frac{1}{\left[ H^{+} \right]^{m}} \right) \right)$$

$$E_{\mathrm{cell}}=E_{\mathrm{cell}}^{^{\circ}}-\left( \frac{2.303RT}{\mathrm{nF}}\log_{10} \left( \frac{\left[ B \right]^{b}}{\left[ A \right]^{a}} \right)+\frac{2.303RT}{\mathrm{nF}}\log_{10} \left( \left[ H^{+} \right]^{-m} \right) \right)$$

$$\mathrm{Using}\log_{10} x^{z}=z\log_{10} x$$

$$E_{\mathrm{cell}}=E_{\mathrm{cell}}^{^{\circ}}-\left( \frac{2.303RT}{\mathrm{nF}}\log_{10} \left( \frac{\left[ B \right]^{b}}{\left[ A \right]^{a}} \right)-\frac{2.303RTm}{\mathrm{nF}}\log_{10} \left[ H^{+} \right] \right)$$

$$Using pH=-\log_{10} \left[ H^{+} \right]$$

$$E_{\mathrm{cell}}=E_{\mathrm{cell}}^{^{\circ}}-\left( \frac{2.303RT}{\mathrm{nF}}\log_{10} \left( \frac{\left[ B \right]^{b}}{\left[ A \right]^{a}} \right)+\frac{2.303RTm}{\mathrm{nF}}\mathrm{pH} \right)$$

$$E_{\mathrm{cell}}=E_{\mathrm{cell}}^{^{\circ}}-\frac{2.303RT}{\mathrm{nF}}\log_{10} \left( \frac{\left[ B \right]^{b}}{\left[ A \right]^{a}} \right)-\frac{2.303RTm}{\mathrm{nF}}\mathrm{pH}$$
